# Sarcopenia is attenuated by TRB3 knockout in aging mice via the alleviation of atrophy and fibrosis of skeletal muscles

**DOI:** 10.1002/jcsm.12560

**Published:** 2020-02-25

**Authors:** Guo‐Kai Shang, Lu Han, Zhi‐Hao Wang, Ya‐Peng Liu, Sen‐Bo Yan, Wen‐Wen Sai, Di Wang, Yi‐Hui Li, Wei Zhang, Ming Zhong

**Affiliations:** ^1^ The Key Laboratory of Cardiovascular Remodeling and Function Research, Chinese Ministry of Education, Chinese National Health Commission and Chinese Academy of Medical Sciences, The State and Shandong Province Joint Key Laboratory of Translational Cardiovascular Medicine, Department of Cardiology Qilu Hospital of Shandong University Jinan Shandong China; ^2^ Department of General Practice Qilu Hospital of Shandong University Jinan China; ^3^ Department of Geriatric Medicine Qilu Hospital of Shandong University, Key Laboratory of Cardiovascular Proteomics of Shandong Province Jinan China; ^4^ Department of Critical Care Medicine Qilu Hospital of Shandong University Jinan China

**Keywords:** Aging, TRB3, Exercise capacity, Atrophy, Fibrosis, Sarcopenia

## Abstract

**Background:**

Sarcopenia causes several adverse events in elderly people. Muscle fibre atrophy and interstitial fibrosis are the main histopathological changes in sarcopenia and account for decreased muscle function. Tribbles homologue 3 (TRB3) was previously reported to exhibit age‐related expression and play a vital role in cell proliferation, differentiation, and fibrosis. We aimed to investigate how TRB3 affects sarcopenia.

**Methods:**

Wild‐type and TRB3 knockout C57/BL6J mice were randomly divided into young and old groups. Exercise capacity was evaluated, and single‐muscle function was detected by electrophysiological techniques, after which the mice were sacrificed to collect their gastrocnemius muscles for assessment of atrophy and fibrosis by histopathological and molecular biological methods. TRB3 expression, autophagy level, and MAPK signalling pathway activity were evaluated through western blotting. The interaction of TRB3 with P62 and the association between TRB3 and the MAPK signalling pathway were detected by co‐immunoprecipitation.

**Results:**

In aged mice, exercise capacity and cross‐sectional area of skeletal muscle fibres were decreased significantly, whereas TRB3, atrophy‐related markers atrogin 1 and MuRF 1, and interstitial fibrosis, including collagen volume fraction, contents of collagens I and III, and ratio of collagens I to III, were increased significantly (*P* < 0.05 for all). Following TRB3 knockout, the cross‐sectional area of muscle fibres, mainly fast fibres, was elevated (*P* < 0.05 for both), the atrogin 1 expression was decreased (*P* = 0.0163), and the corresponding tetanic force of fast muscles was increased (*P* = 0.0398). Conversely, interstitial fibrosis was substantially decreased and exercise capacity was significantly increased in the knockout mice. In terms of the underlying mechanisms, the autophagy receptor p62 was markedly increased and the MAPK signalling pathway was activated in aged skeletal muscles, which might be attributed to the interaction of TRB3 with p62 and MAPKKs, including MEK1/MEK2, MEK3/MEK6, and MEK4/MKK4. Notably, TRB3 knockout reduced the accumulation of p62 and LC3 (*P* < 0.05 for both), decreased the phosphorylation of JNK (*P* = 0.0015), and increased p38 phosphorylation (*P* = 0.0021).

**Conclusions:**

TRB3 knockout in mice attenuated muscle fibre atrophy and reduced skeletal muscle fibrosis by increasing autophagy and inhibiting the MAPK signalling pathway. Correspondingly, in aged knockout mice, exercise capacity was improved. Interfering with TRB3 expression in aged skeletal muscles may serve as a target for the prevention and treatment of age‐related sarcopenia.

## Introduction

Sarcopenia is an age‐related syndrome characterized by progressive and extensive loss of skeletal muscle mass and strength, and the major pathological manifestations of sarcopenia are a decrease in muscle fibres and an increase in interstitial fibrous and adipose tissues.^1^ Muscle tissue content is reported to gradually decrease from the age of 35 in humans, with the content being reduced by up to 30% in people in their 50s to 80s, and sarcopenia affects almost all elderly people.^2^ Sarcopenia triggers numerous adverse events, including falls, loss of function, weakness, and even death.^3^ In this century, considerable socio‐economic burden has been incurred because of population aging, and consequently, substantial research attention has been devoted towards investigating the potential mechanisms of and effective intervention targets for sarcopenia.

Atrophy of muscle fibres and accumulation of interstitial fibrous tissue are major histopathological changes in sarcopenia. Both the size and the number of skeletal muscle fibres decrease in elderly people, and the decrease occurs earlier in fast (type II) muscle fibres than slow (type I) muscle fibres^4^, ^5^, ^6^, ^7^ and contributes to the recession of exercise capacity, particularly the generation of explosive force. Furthermore, interstitial fibrosis is clearly observed in aged skeletal muscles,^8^, ^9^ and the increase in fibrous tissue leads to enhanced muscle stiffness, which restricts muscle stretching and contraction and thereby lowers the exercise capacity of elderly people. Moreover, the deposition of fibrous tissue might interfere with the interactions between satellite cells and surrounding cells and thus lead to impaired muscle regeneration.^10^, ^11^ However, the mechanisms underlying muscle fibre atrophy and fibrous tissue accumulation remain unclear.

TRB3 was previously reported to exhibit age‐related expression^12^ and play a vital role in cell proliferation, differentiation, and fibrosis. It has been demonstrated that TRB3 caused muscle fibre atrophy and functional recession by negatively modulating protein turnover in the condition of food deprivation^13^, ^14^ and could inhibit the myogenic differentiation of C2C12 cells.^15^ TRB3 was also found to induce interstitial fibrosis in various diseases such as systemic sclerosis, diabetic nephropathy, and diabetic cardiomyopathy.^16^, ^17^, ^18^ However, the role of TRB3 in aged skeletal muscles remains to be elucidated. Herein, we investigated the effects of TRB3 on skeletal muscle fibre atrophy and fibrosis in naturally aging wild‐type (WT) and TRB3 knockout mice.

## Materials and methods

### Animals

TRB3 knockout (TRB3^−/−^) mice [background: C57/BL6J (hereafter ‘C57’)] were donated by Prof. Laurie J. Goodyear of Joslin Diabetes Research Center, Harvard University, USA. The Material Transfer Agreements were approved and uploaded as attachments. Four‐week‐old male WT C57 mice were purchased from Beijing HFK Biotechnology. All mice were bred and raised in the animal room of the cardiovascular laboratory at Qilu Hospital of Shandong University. Breeding mice were housed three per cage (two female and one male), whereas experimental mice were housed five per cage. All mice were housed at 22 °C under a 12/12 h light–dark cycle with adequate food and water. The mice were randomized into four groups (nine mice per group): WT young group, WT old group, TRB3^−/−^ young group, and TRB3^−/−^ old group; the young‐group and old‐group mice were raised until they were 3 and 18 months old, respectively, and used in the various assays described in the succeeding text at the end of the experiment. All animal studies were approved by the appropriate ethics committee and performed in accordance with the ethical standards specified in the 1964 Declaration of Helsinki and its later amendments. All experiments were approved by the ethics boards of Qilu Hospital of Shandong University.

### Forelimb grip strength test

Forelimb grip strength was measured using an electronic dynamometer (Handpi HP‐5N, China). Taking advantage of the instinct of a mouse to grab what they could while suspended, we trained the mouse to grasp the horizontal bar that was attached to the dynamometer. We gently pulled the mouse backwards in a horizontal direction parallel to the dynamometer and forced the mouse to fight the pull. As the force we applied to the mouse gradually increased, the mouse could not fight the force, it would release the bar. The force we gently applied to the mouse was equal to the force the mouse applied to the horizontal bar. Forelimb grip strength of each mouse was tested thrice, and the three measured values were recorded and averaged.

### Hanging grid test

In this assay, inverted hanging time was measured. A 45 × 45 cm grid (bar thickness, 2 mm; mesh, 18 mm) was placed on a 55‐cm‐high frame, and a 5‐cm‐thick cushion was placed under the grid. The distance between the grid and the cushion was 50 cm. We placed each mouse at the centre of the grid and then turned the grid upside down with the mouse head declining first. Hanging time was recorded as the time until the mice fell. Each mouse was tested thrice with a >30 min interval between tests, and the hanging time was recorded and averaged.

### Exhaustive running test

Exhaustive running time was measured using a treadmill. Two days before the test, the mice were forced to perform adaptive exercise on the treadmill twice at a speed of 10 m/min and a slope angle of 0°. On the third day, the running test was conducted. The running was started at 13 m/min with a slope angle of 0°, and then the speed and slope angle were increased by 2 m/min and 2° every 3 min until they reached 39 m/min and 14°, respectively. When the mouse being tested did not return to the track for >20 s and concomitantly exhibited a markedly diminished response to external stimuli, the mouse was moved out from the treadmill and the exhaustive running time was recorded.

### Single‐muscle function

Mice were anaesthetized by intraperitoneally injecting pentobarbital sodium (80 mg/kg) and fixed on a heating pad to maintain body temperature. Extensor digitorum longus (EDL) muscle was exposed, and the distal tendon was tied with a surgical suture, and the tendon was dissected at the distal end of the knot. Next, the muscle was separated up to the proximal muscle attachment site, and the other end of the suture was tied to a force transducer and maintained at a low base tension. The muscle was connected to an isolated pulse stimulator (A‐M SYSTEMS MODEL 2100, USA) by a platinum wire and stimulated at 20 V/cm. Analogue signals were converted using an A/D converter and recorded using LabChart 5.0 (ADInstruments). Stimulation with a 5 ms square wave was used while gradually increasing the base tension until the optimal base tension was discovered. With the optimal base tension being used, 5 ms pulses at 0.2 Hz were applied thrice to determine the maximal isometric twitch force. Maximal isometric tetanic force was detected thrice by stimulating the muscle with 5 ms pulses at 100 Hz for 300 ms with a 60 s interval between stimulations; subsequently, the muscle was isolated and transected, and the maximal cross‐sectional area (CSA) was determined using a stereoscope (Nikon SMZ25) and Image‐Pro Plus.

### Tissue processing

Mice were weighed and then anaesthetized by intraperitoneally injecting pentobarbital sodium (80 mg/kg). Tibia length was measured. Bilateral gastrocnemius muscles were collected and weighed, one muscle was frozen at −80 °C after liquid nitrogen treatment, and the other was fixed with 4% paraformaldehyde, dehydrated with a gradient ethanol series, and embedded with paraffin for preparing 5 μm sections.

### Haematoxylin–eosin staining

After slides were dewaxed and hydrated using a dewaxing solution and gradient ethanol series, they were soaked in haematoxylin for 3 min, then subjected to 5 s differentiation with 1% hydrochloric acid ethanol to turn the nucleus blue in colour, and then lastly soaked in eosin for 2 min to stain the cytoplasm red. After dehydration with ethanol and clearing in a dewaxing solution, the slides were mounted. Images were acquired using an Olympus DP72 digital imaging system (Olympus Corporation, Tokyo, Japan), and the CSA of muscle fibres was calculated using Image‐Pro Plus.

### Masson staining

After dewaxing and hydration, slides were sequentially soaked in ponceau magenta for 10 min, 0.2% glacial acetic acid for 1 min, phosphomolybdic acid for 1 min, and 0.2% glacial acetic acid for 1 min to turn the cytoplasm red in colour. Moreover, slides were treated with aniline blue for 30 s and soaked with 0.2% glacial acetic acid for 1 min to stain the fibrous tissue blue. The slides were subsequently dehydrated using ethanol, cleared in a dewaxing solution, and mounted. After image acquisition, the collagen volume fraction (ratio of blue dye area to red dye area) was calculated using Image‐Pro Plus.

### Sirius Red staining

Slides were dewaxed and hydrated, soaked in Sirius Red dye solution for 1 h, washed with running water for 30 s, dehydrated with ethanol, cleared in a dewaxing solution, and mounted. Images were captured, and collagen volume fraction was calculated using Image‐Pro Plus.

### Immunohistochemical staining

After slides were dewaxed and hydrated, antigens were retrieved using a citric acid buffer in a microwave oven for 20 min [for type I collagen, type III collagen, and fast myosin heavy chain (MyHC)] or using proteinase K at 37 °C for 30 min (for slow MyHC). Endogenous peroxidases were inactivated by treating with 3% hydrogen peroxide for 10 min, and 5% bovine serum albumin was added to block non‐specific binding. Next, the tissue sections were incubated overnight at 4 °C with primary antibodies (from Abcam, UK): Anti‐Collagen I (ab34710), Anti‐Collagen III (ab7778), Anti‐Fast Myosin Skeletal Heavy Chain (ab51263), and Anti‐Slow Myosin Skeletal Heavy Chain (ab11083). Subsequently, the sections were incubated with an enhancing solution at room temperature for 15 min and then with secondary antibodies at 37 °C for 15 min. Sections were washed with phosphate‐buffered saline for 3 times between each of the aforementioned steps. Lastly, 3,3′‐diaminobenzidine (DAB) solution (Wuhan Servicebio Technology G1212, China) was added, and the sections were examined under a microscope. When the target proteins turned yellow, the slides were transferred to distilled water, after which they were soaked in haematoxylin for 3 min, washed in running water for 30 s, subjected to differentiation in 1% hydrochloric acid ethanol for 1 s, dehydrated with ethanol, cleared in a dewaxing solution, and mounted. After acquisition, images were analysed using Image‐Pro Plus. The content of collagens I and III was represented by their integrated optical density value; the size of fast and slow muscle fibres was represented by their CSA (area divided by number of cells in positive region); and the relative content of fast and slow muscle fibres was represented by the number of slow‐MyHC‐positive cells divided by the number of fast‐MyHC‐positive cells.

### Western blotting

Proteins were extracted from gastrocnemius muscles, separated on 10–12% sodium dodecyl sulfate–polyacrylamide gels, and transferred onto PVDF membranes (Millipore IPVH304F0, USA), which were soaked in Tris‐buffered saline–Tween (TBST) solution containing 5% bovine serum albumin (room temperature, 1 h) to block non‐specific binding; subsequently, the membranes were exposed to primary antibodies: Anti‐Collagen I (Abcam ab34710), Anti‐Collagen III (Abcam ab7778), Anti‐Fbx32 (Abcam ab168372), Anti‐TRIM63 (Proteintech 55456‐1‐AP, USA), Anti‐GAPDH (Proteintech 66009‐1‐Ig), Anti‐CDKN2A/p16INK4a (Abcam ab211542), Anti‐P21 (Abcam ab188224), Anti‐P53 (Abcam ab26), Anti‐β‐galactosidase (Proteintech 15518‐1‐AP), Anti‐TRB3 (Proteintech 13300‐1‐AP), Anti‐LC3B (Abcam ab192890), Anti‐SQSTM1/p62 (Abcam ab109012), Anti‐JNK1 + JNK2 + JNK3 (Abcam ab208035), Anti‐JNK1 + JNK2 + JNK3 (phospho‐T183 + T183 + T221) (Abcam ab124956), Anti‐ERK1 + ERK2 (Abcam ab184699), Anti‐Erk1 (pT202/pY204) + Erk2 (pT185/pY187) (Abcam ab76299), Anti‐p38 (Abcam ab170099), Anti‐p38 (phospho‐T180 + Y182) (Abcam ab195049). After staining overnight at 4 °C, the membranes were washed thrice with TBST and incubated with horseradish peroxidase (HRP)‐labeled anti‐rabbit (ZSGB‐BIO ZB‐2305, China) or anti‐mouse (ZSGB‐BIO ZB‐2301) secondary antibodies (as appropriate) at room temperature for 90 min. After washing thrice more with TBST, an enhanced chemiluminescence (ECL) reagent (Millipore WBKLS0500) was added, and then images were acquired and quantified using ImageJ.

### Co‐immunoprecipitation

Total proteins were extracted from gastrocnemius muscles of young and old WT mice by using an immunoprecipitation kit (Proteintech KIP2). Protein concentration was determined using a BCA Protein Assay Kit (Solarbio PC0020, China). After removing endogenous antibodies by using Protein A Sepharose beads, proteins were incubated (overnight at 4 °C) with anti‐TRB3 antibody (Proteintech 13300‐1‐AP) or IgG (Proteintech B900610), and then the antibodies and the captured proteins were precipitated using Protein A Sepharose beads and eluted using an elution buffer. The eluted proteins were separated on sodium dodecyl sulfate–polyacrylamide gels, transferred onto PVDF membranes (Millipore IPVH304F0), and probed with primary antibodies (from Abcam): Anti‐MEK1 + MEK2 (ab178876), Anti‐MEK3 + MEK6 (ab200831), Anti‐MEK4/MKK4 (ab33912), Anti‐MKK7 (ab52618), and Anti‐SQSTM1/p62 (ab109012). After incubation at 4 °C overnight, the membranes were exposed to HRP‐mouse anti‐rabbit IgG light chain‐specific secondary antibody (Proteintech SA00001‐7L), and then immunoreactive bands were visualized using the Millipore ECL reagent (WBKLS0500).

### Statistical analysis

Data are represented as means ± SEM. SPSS 19.0 and GraphPad Prism 6.0 were used for statistical analysis and image presentation. Comparisons between two groups were performed using unpaired *t*‐tests, whereas comparisons among four groups were performed using two‐way analysis of variance and Tukey's test. Correlation between TRB3 protein expression and skeletal muscle fibre size or fibrous tissue content was evaluated using Pearson's correlation coefficient. *P* < 0.05 was considered significant.

## Results

### Establishment of natural aging animal model

**Figure 1:**
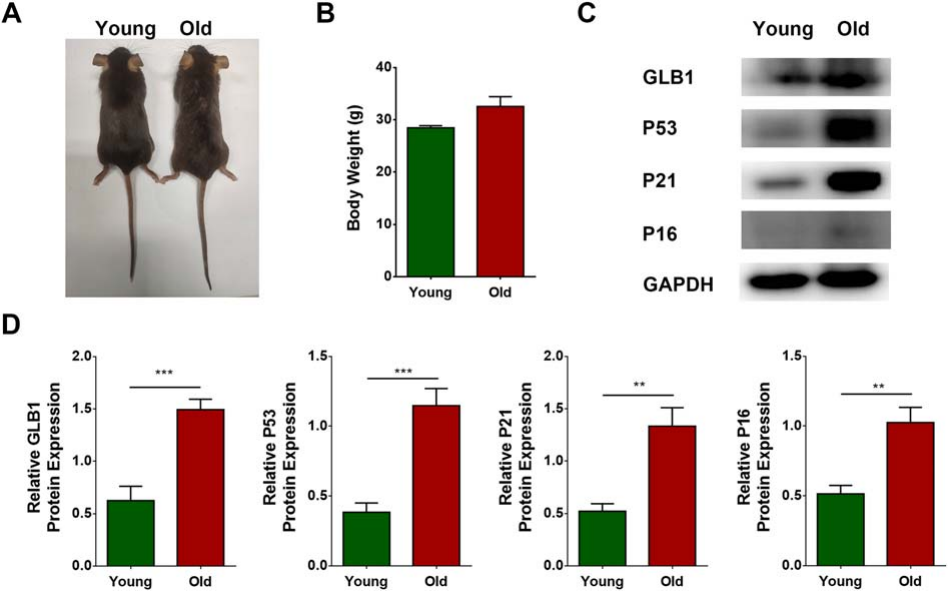
Establishment of natural aging animal model. Wild‐type C57 mice were divided into young and old groups. (A) Photograph of mice. (B) Body weight (g). (C) Expression of GLB1, p53, p21, and p16 detected through western blotting; GAPDH: internal reference. (D) Relative protein expression levels of GLB1, p53, p21, and p16. *N* = 6; ^**^ *P* < 0.01 and ^***^ *P* < 0.001.

A natural aging animal model was established by raising WT C57 mice until they were 18 months old. As compared with young control mice, which featured bushy shiny hair and were responsive and quick, old mice showed sparse bleak hair and were dull and slow (*Figure* 1A), and the old mice were slightly heavier than the young mice (*P* > 0.05) (*Figure* 1B). Western blotting (*Figure* 1C) revealed that the relative contents of the age‐related markers β‐galactosidase (GLB1), p53, p21, and p16 in muscles were significantly higher in old mice than in young mice (*P* = 0.0004, 0.0003, 0.0016, and 0.0022, respectively) (*Figure* 1D). These changes suggested that we had successfully established a natural aging animal model.

### Exercise capacity was markedly decreased in aged mice

**Figure 2:**
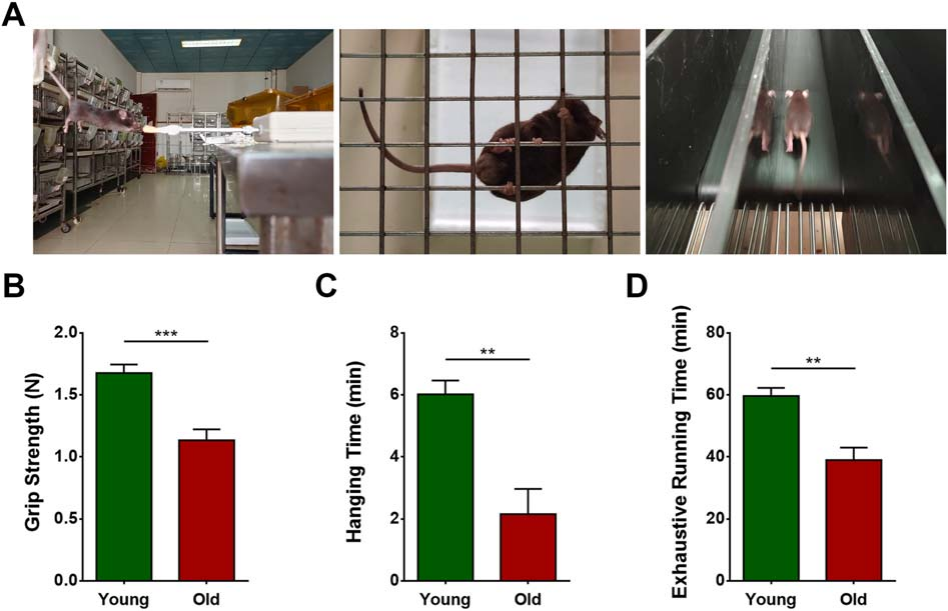
Exercise capacity was markedly decreased in aged mice. Wild‐type C57 mice were divided into young and old groups. (A) Forelimb grip strength test, hanging grid test, and exhaustive running test. (B) Forelimb grip strength (N). (C) Inverted hanging time (min). (D) Exhaustive running time (min). *N* = 6; ^**^ *P* < 0.01 and ^***^ *P* < 0.001.

The effects of aging on exercise capacity were tested at the end of the experiment. Forelimb grip strength, inverted hanging time, and treadmill exhaustive running time were significantly lower in the old group than in the young group (*P* = 0.0007, 0.0019, and 0.0015, respectively) (*Figure* 2).

### Histopathological changes of skeletal muscles in aged mice

#### Skeletal muscle fibres, particularly fast muscle fibres, were overtly atrophied in aged mice

**Figure 3:**
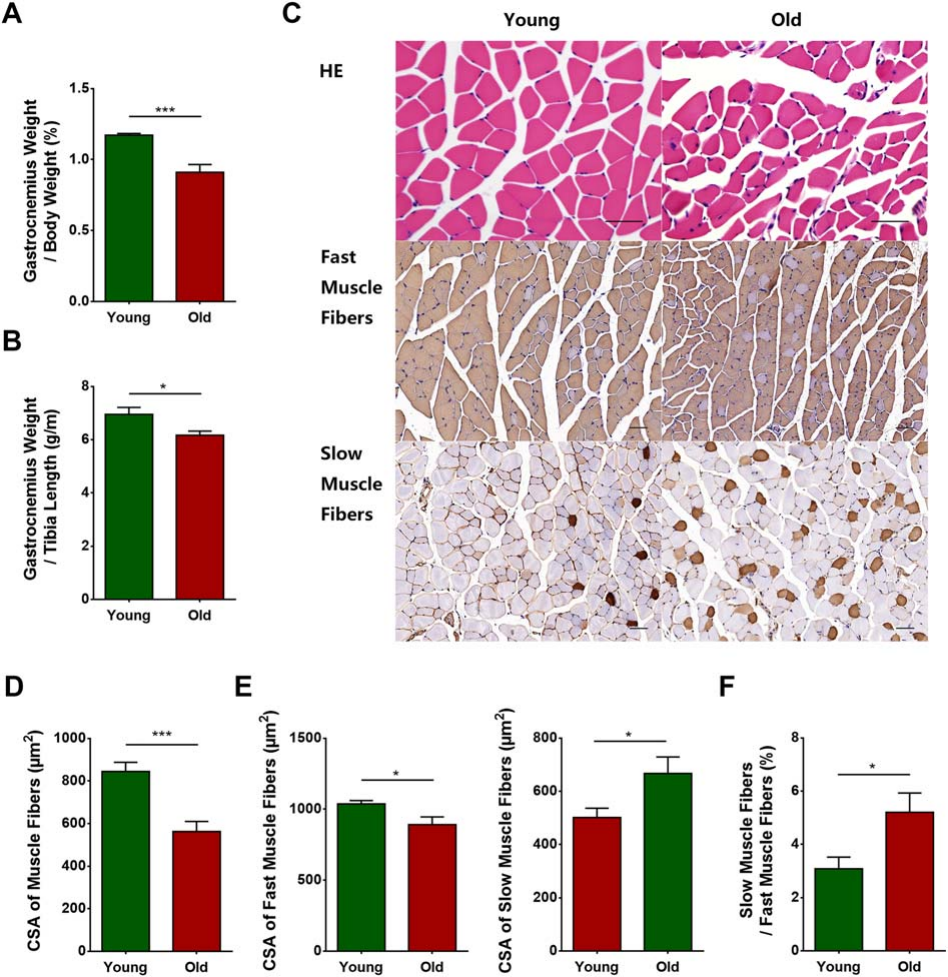
Skeletal muscle fibres, particularly type II muscle fibres, were atrophied in aged mice. Wild‐type C57 mice were divided into young and old groups. (A) Percentage of gastrocnemius weight in body weight (%). (B) Ratio of gastrocnemius weight to tibia length (g/m). (C) Haematoxylin–eosin (HE) and immunohistochemical staining of fast and slow myosin heavy chain. (D) Cross‐sectional area (CSA) of muscle fibres (μm^2^). (E) CSA of fast and slow muscle fibres (μm^2^). (F) Percentage of the number of slow muscle fibres in the number of fast muscle fibres (%). *N* = 6; ^*^ *P* < 0.05 and ^***^ *P* < 0.001. Bar = 50 μm.

We next examined how aging affected skeletal muscle atrophy in mice: we divided the weights of bilateral gastrocnemius muscles by body weight and the weight of unilateral gastrocnemius muscle by tibia length. The percentage of gastrocnemius weight in body weight and the ratio of gastrocnemius weight to tibia length were significantly lower in the old group than in the control group (*P* = 0.0008 and 0.0280, respectively) (*Figure* 3A and 3B).

The results of haematoxylin–eosin staining showed that aged skeletal muscle fibres exhibited clear variations in size and arrangement (*Figure* 3C), and the CSA of muscle fibres was significantly decreased in aged skeletal muscles relative to control (*P* = 0.0009) (*Figure* 3D). To ascertain whether aging produced distinct effects on fast and slow muscle fibres, we performed immunohistochemical staining for slow and fast MyHC (*Figure* 3C). As compared with the corresponding CSA in the young group, the CSA of fast and slow muscle fibres in the aged group was respectively decreased (*P* = 0.0326) and increased (*P* = 0.0418) (*Figure* 3E). Moreover, the percentage of slow in fast muscle fibres was increased significantly in the aged group (*P* = 0.0308) (*Figure* 3F).

#### Interstitial fibrosis was clearly evident in aged skeletal muscles

**Figure 4:**
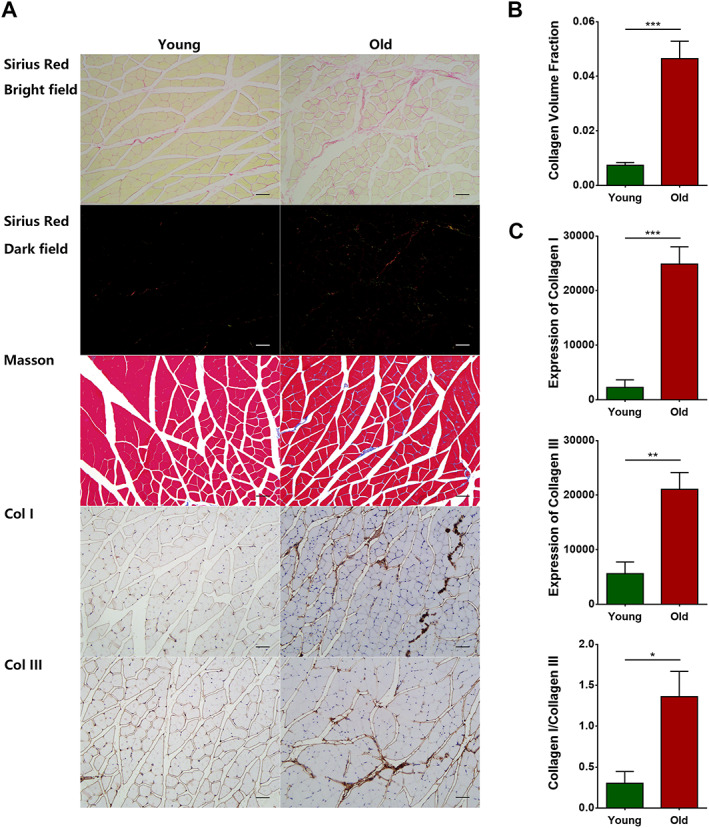
Interstitial fibrosis was clearly evident in aged skeletal muscles. Wild‐type C57 mice were divided into young and old groups. (A) Bright‐field and dark‐field imaging of Sirius Red staining, Masson staining, and immunohistochemical staining of collagen I and collagen III. (B) Collagen volume fraction. (C) Analyses of collagen I and collagen III expression. *N* = 6; ^*^ *P* < 0.05, ^**^ *P* < 0.01, and ^***^ *P* < 0.001. Bar = 50 μm.

Sirius Red staining and Masson staining revealed an increase in fibrous tissue in aged skeletal muscles (*Figure* 4A). The collagen volume fraction was significantly higher in the old group than in the young group (*P* = 0.0001) (*Figure* 4B). Immunohistochemical staining results further showed that both collagen I and collagen III were increased in aged skeletal muscles (*P* < 0.0001 and *P* = 0.002, respectively) (*Figure* 4A and 4C), and the ratio of the content of collagen I to collagen III was also increased (*P* = 0.0111) (*Figure* 4C).

### TRB3 expression was related to atrophy and fibrosis of aged skeletal muscles

#### TRB3 was highly expressed in aged skeletal muscles

**Figure 5:**
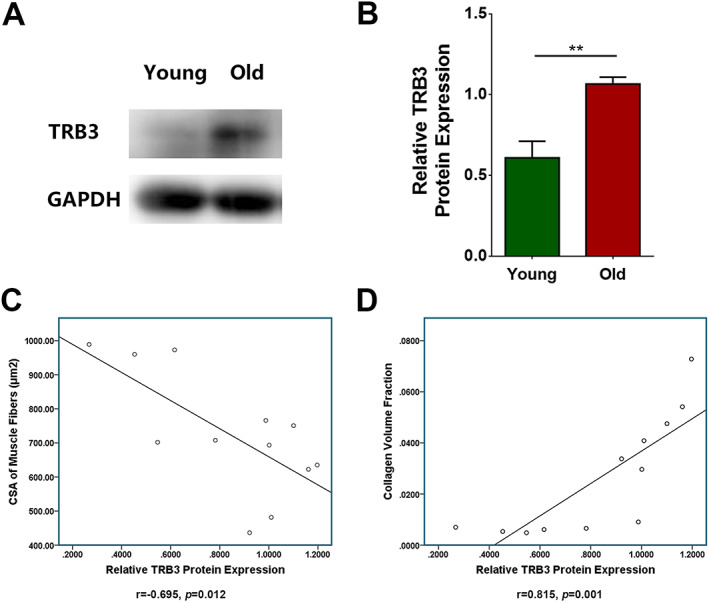
TRB3 expression was related to atrophy and fibrosis of aged skeletal muscles. Wild‐type C57 mice were divided into young and old groups. (A) Detection of TRB3 expression by means of western blotting; GAPDH: internal reference. (B) Relative expression of TRB3 protein. (C) Correlation between cross‐sectional area (CSA) of skeletal muscle fibres and TRB3 protein expression. (D) Correlation between collagen volume fraction and TRB3 protein expression. *N* = 6; ^**^ *P* < 0.01.

Western blotting results showed that TRB3 expression was significantly higher in aged skeletal muscles than in young skeletal muscles (*P* = 0.0022) (*Figure* 5A and 5B).

#### TRB3 expression was related to skeletal muscle fibre atrophy

We evaluated the correlation between TRB3 protein expression and skeletal muscle fibre size by determining Pearson's correlation coefficient, which revealed that the CSA of skeletal muscle fibres was negatively related to TRB3 expression (*r* = −0.695, *P* = 0.012) (*Figure* 5C).

#### TRB3 expression was related to skeletal muscle fibrosis

Pearson's correlation coefficient was also used to evaluate the correlation between TRB3 protein expression and fibrous tissue content in skeletal muscles. The results showed that the collagen volume fraction of skeletal muscle fibres was positively related to TRB3 expression (*r* = 0.815, *P* = 0.001) (*Figure* 5D).

### Effects of TRB3 knockout on aged skeletal muscles

#### TRB3 knockout alleviated skeletal muscle fibre atrophy in aged mice

**Figure 6:**
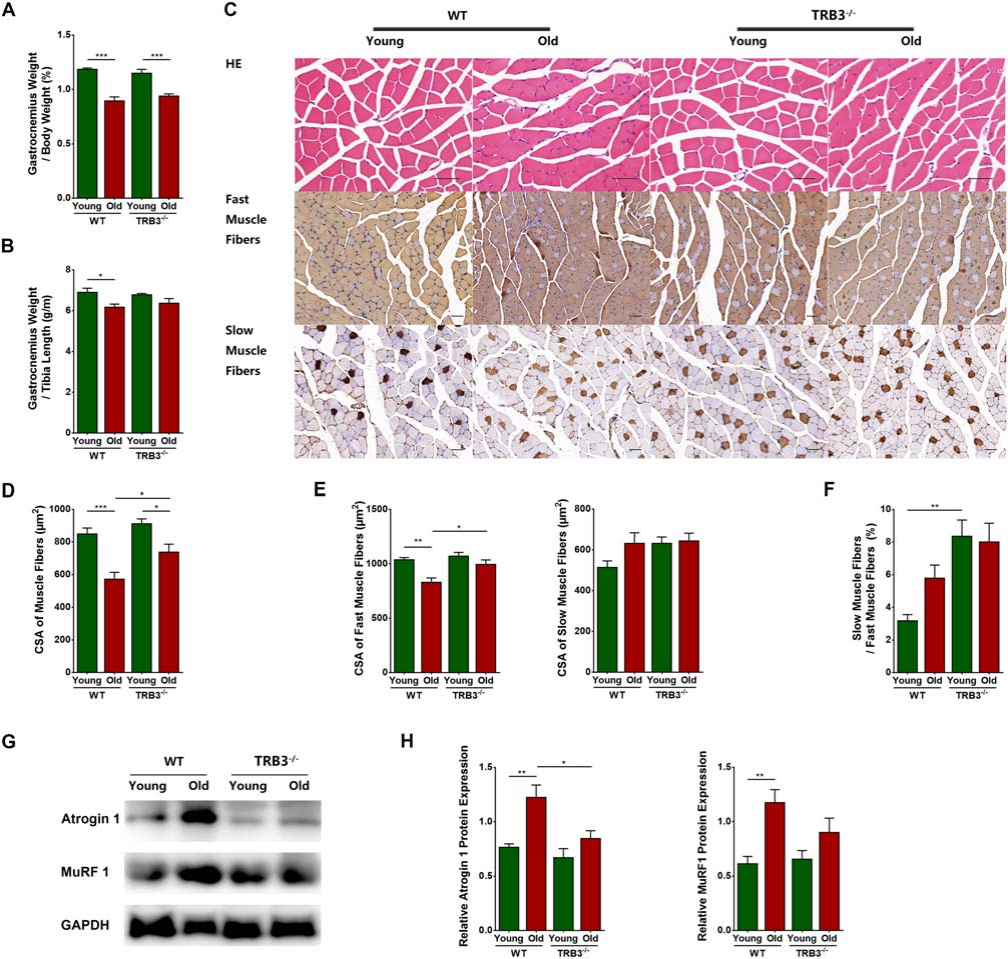
TRB3 knockout alleviated skeletal muscle fibre atrophy in aged mice. Mice were divided into wild‐type (WT) young group, WT old group, TRB3^−/−^ young group, and TRB3^−/−^ old group. (A) Percentage of gastrocnemius weight in body weight (%). (B) Ratio of gastrocnemius weight to tibia length (g/m). (C) Haematoxylin–eosin (HE) and immunohistochemical staining of fast and slow myosin heavy chain. (D) Cross‐sectional area (CSA) of muscle fibres (μm^2^). (E) CSA of fast and slow muscle fibres (μm^2^). (F) Percentage of the number of slow muscle fibres in the number of fast muscle fibres (%). (G) Detection of atrogin 1 and MuRF1 expression through western blotting; GAPDH: internal reference. (H) Relative protein expression levels of atrogin 1 and MuRF1. *N* = 6–9; ^*^ *P* < 0.05, ^**^ *P* < 0.01, and ^***^ *P* < 0.001. Bar = 50 μm.

Young and old TRB3 knockout mice were examined in comparison with young and old WT mice. The percentage of gastrocnemius weight in body weight measured for the four groups revealed that the percentage was lower in the WT old group than in the WT young group (*P* < 0.0001) and in the TRB3^−/−^ old group than in the TRB3^−/−^ young group (*P* = 0.0001). The percentage did not differ between the two young groups (*P* > 0.05), but the percentage of the TRB3^−/−^ old group exhibited an increasing trend as compared with that of the WT old group (*P* > 0.05) (*Figure* 6A). Next, we calculated the ratio of gastrocnemius weight to tibia length; the WT old group showed a significant decrease in the ratio relative to the WT young group (*P* = 0.0443), and the TRB3^−/−^ old group exhibited a non‐significant increase relative to the WT old group (*P* > 0.05). No significant changes were measured between the two young groups and between the two TRB3^−/−^ groups (*P* > 0.05 for both) (*Figure* 6B).

Haematoxylin–eosin staining results showed marked variations in skeletal muscle fibre size and arrangement in the WT old group but a comparatively more uniform arrangement in the TRB3^−/−^ old group (*Figure* 6C). Moreover, muscle fibre CSA was significantly lower in the WT old group than in the WT young group (*P* = 0.0001) and in the TRB3^−/−^ old group than in the TRB3^−/−^ young group (*P* = 0.0187). The CSA did not differ between the two young groups (*P* > 0.05), but the CSA of muscle fibres in the TRB3^−/−^ old group was higher than that in the WT old group (*P* = 0.0326) (*Figure* 6D).

Immunohistochemical staining was next performed to investigate the effects of TRB3 on different types of muscle fibres (*Figure* 6C). The CSA of fast muscle fibres was decreased significantly in the WT old group relative to that in the WT young group (*P* = 0.0012), decreased non‐significantly in the TRB3^−/−^ old group as compared with that in the TRB3^−/−^ young group (*P* > 0.05), and increased significantly in the TRB3^−/−^ old group relative to that in the WT old group (*P* = 0.0146). Conversely, the CSA of slow muscle fibres was increased non‐significantly in the WT old group and the TRB3^−/−^ young group as compared with that in the WT young group but showed no significant differences between either the two old groups or the two TRB3^−/−^ groups (*P* > 0.05 for all) (*Figure* 6E). Lastly, the percentage of slow in fast muscle fibres was increased non‐significantly in the WT old group (*P* > 0.05) and significantly in the TRB3^−/−^ young group (*P* = 0.0017) relative to that in the WT young group, and the percentage was increased non‐significantly in the TRB3^−/−^ old group (*P* > 0.05) as compared with that in the WT old group (*Figure* 6F).

We next used western blotting to detect the expression of muscle atrophy‐related markers (*Figure* 6G). Expression of atrogin 1 and MuRF1 was significantly higher in the WT old group than in the WT young group (*P* = 0.0033 and 0.0048, respectively) but showed no notable difference between either the two young groups or the two TRB3^−/−^ groups (*P* > 0.05 for all). However, as compared with the expression in the WT old group, atrogin 1 showed a significant decrease (*P* = 0.0163) and MuRF1 showed a non‐significant decrease (*P* > 0.05) in the TRB3^−/−^ old group (*Figure* 6H).

#### TRB3 knockout reduced skeletal muscle interstitial fibrosis in aged mice

**Figure 7:**
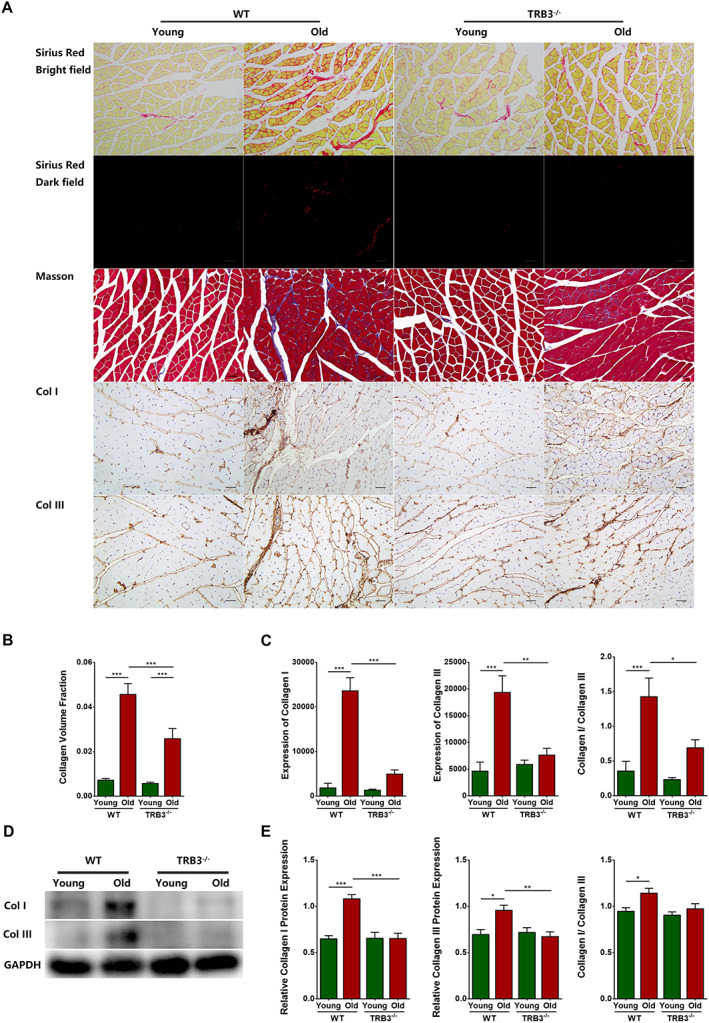
TRB3 knockout reduced skeletal muscle interstitial fibrosis in aged mice. Mice were divided into WT young group, WT old group, TRB3^−/−^ young group, and TRB3^−/−^ old group. (A) Bright‐field and dark‐field imaging of Sirius Red staining, Masson staining, and immunohistochemical staining of collagen I and collagen III. (B) Collagen volume fraction. (C) Immunohistochemical analyses of collagen I and collagen III expression. (D) Collagen I and collagen III expression detected through western blotting; GAPDH: internal reference. (E) Relative expression of collagen I and collagen III. *N* = 6–9; ^*^ *P* < 0.05, ^**^ *P* < 0.01, and ^***^ *P* < 0.001. Bar = 50 μm.

Sirius Red staining and Masson staining of muscles from the four groups (*Figure* 7A) revealed that the collagen volume fraction was significantly higher in the WT old group than in the WT young group (*P* < 0.0001). A similar change but with a comparatively lesser increase was measured in the TRB3^−/−^ old group relative to the TRB3^−/−^ young group (*P* = 0.0005), and no significant difference was found between the two young groups (*P* > 0.05); by contrast, the collagen volume fraction was significantly lower in the skeletal muscles of the TRB3^−/−^ old group than of the WT old group (*P* = 0.0009) (*Figure* 7B).

Immunohistochemical staining for different types of collagen (*Figure* 7A) revealed that collagen I and collagen III showed significant increases in their content in the WT old group as compared with the level in the WT young group (*P* < 0.0001 for both) but exhibited non‐significant increases in the TRB3^−/−^ old group relative to the TRB3^−/−^ young group (*P* > 0.05 for both). No significant differences were found between the two young groups (*P* > 0.05 for both), but both collagen I and collagen III were detected at significantly lower levels in the TRB3^−/−^ old group than in the WT old group (*P* < 0.0001 and *P* = 0.0011). Furthermore, the ratio of collagen I to collagen III was increased significantly in the WT old group as compared with that in the WT young group (*P* = 0.0003), increased non‐significantly in the TRB3^−/−^ old group relative to that in the TRB3^−/−^ young group (*P* > 0.05), and decreased significantly in the TRB3^−/−^ old group as compared with that in the WT old group (*P* = 0.0165) (*Figure* 7C).

The aforementioned results were confirmed by performing western blotting analyses (*Figure* 7D): collagen I and collagen III levels were significantly higher in the WT old group than in the WT young group (*P* < 0.0001 and *P* = 0.0124, respectively) and were significantly lower in the TRB3^−/−^ old group than in the WT old group (*P* = 0.0001 and 0.0064). Moreover, the ratio of collagen I to collagen III was increased significantly in the WT old group as compared with that in the WT young group (*P* = 0.0328) and decreased non‐significantly in the TRB3^−/−^ old group relative to that in the WT old group (*P* > 0.05) (*Figure* 7E). These results were largely similar to those of immunohistochemical staining.

#### TRB3 knockout enhanced exercise capacity and single‐muscle function in aged mice

**Figure 8:**
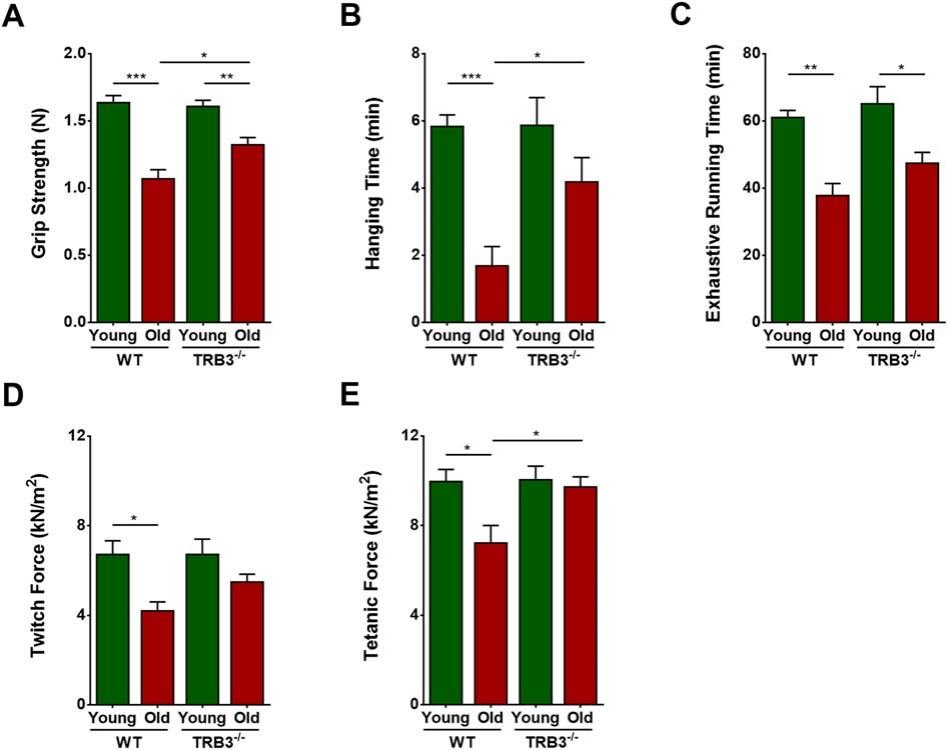
TRB3 knockout enhanced exercise capacity and muscle function in aged mice. Mice were divided into WT young group, WT old group, TRB3^−/−^ young group, and TRB3^−/−^ old group. (A) Forelimb grip strength (N). (B) Inverted hanging time (min). (C) Exhaustive running time (min). (D) Twitch force (kN/m^2^). (E) Tetanic force (kN/m^2^). *N* = 6–9; ^*^ *P* < 0.05, ^**^ *P* < 0.01, and ^***^ *P* < 0.001.

Exercise capacity was tested in the four groups. Forelimb grip strength, inverted hanging time, and treadmill exhaustive running time were significantly lower in the WT old group than in the WT young group (*P* < 0.0001, *P* = 0.0004, and *P* = 0.0010, respectively). Grip strength and exhaustive running time were also significantly lower in the TRB3^−/−^ old group than in the TRB3^−/−^ young group (*P* = 0.0052 and 0.0142, respectively), but hanging time showed a non‐significant decreasing trend (*P* > 0.05). No significant difference in the three measurements was found between the two young groups (*P* > 0.05 for all). However, as compared with the mice in the WT old group, mice in the TRB3^−/−^ old group showed significantly increased grip strength and hanging time (*P* = 0.0151 and 0.0452, respectively) and a tendency of increased exhaustive running time (*P* > 0.05) (*Figure* 8A–8C).

Single‐muscle function was tested in the four groups. EDL twitch force and tetanic force were significantly lower in the WT old group than in the WT young group (*P* = 0.0159 and 0.0219, respectively), but no notable changes were found between either the two young groups or the two TRB3^−/−^ groups (*P* > 0.05 for all). By contrast, twitch force was increased non‐significantly (*P* > 0.05) and tetanic force was increased significantly (*P* = 0.0398) in the TRB3^−/−^ old group as compared with the measured forces in the WT old group.

### Mechanisms underlying the protective effects of TRB3 knockout on skeletal muscles

#### TRB3 knockout increased autophagy in skeletal muscles of aged mice

**Figure 9:**
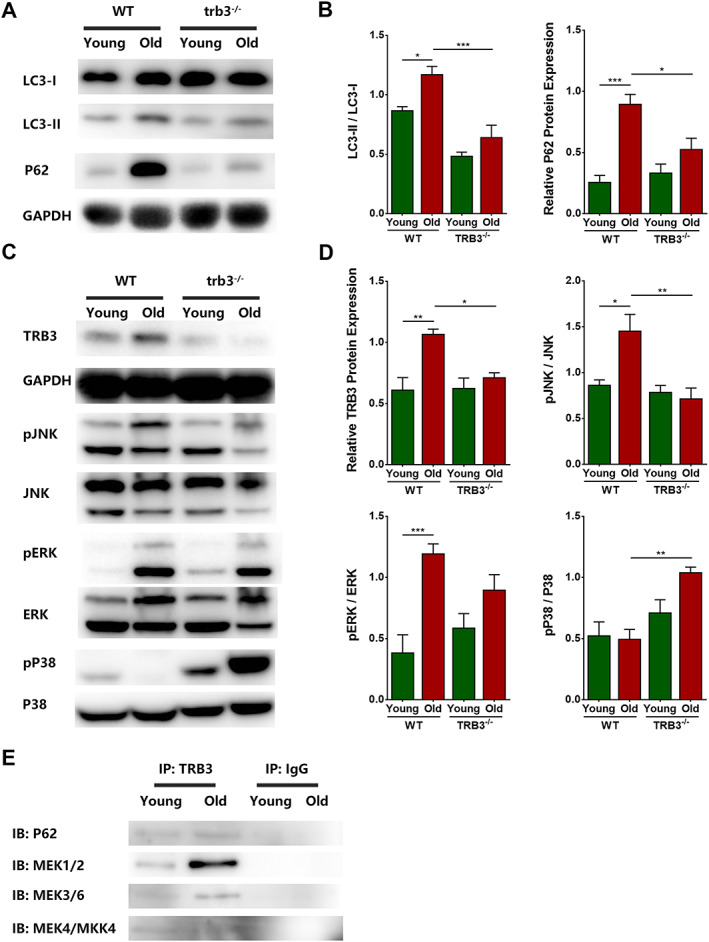
Mechanisms underlying protective effects of TRB3 knockout on skeletal muscles. Mice were divided into WT young group, WT old group, TRB3^−/−^ young group, and TRB3^−/−^ old group. (A) LC3 and p62 expression detected through western blotting; GAPDH: internal reference. (B) Analyses of LC3‐II/LC3‐I and relative p62 protein expression. (C) Levels of TRB3, phospho‐JNK (pJNK), JNK, pERK, ERK, pp38, and p38 detected through western blotting; GAPDH: internal reference. (D) Analyses of TRB3/GAPDH, pJNK/JNK, pERK/ERK, and pp38/p38. (E) Co‐immunoprecipitation analyses of TRB3 interactions with p62 and MAPKKs. *N* = 6; ^*^ *P* < 0.05, ^**^ *P* < 0.01, and ^***^ *P* < 0.001.

LC3 is a biomarker of autophagosomes. The autophagy receptor p62 binds with LC3‐autophagosomes and promotes the degradation of abnormal proteins, LC3, and p62, and the content of p62 is negatively correlated with the level of autophagy. Western blotting results showed that the ratio of LC3‐II to LC3‐I and the p62 content in skeletal muscles were significantly higher in the WT old group than in the WT young group (*P* = 0.0312 and 0.0001, respectively) but were significantly lower in the TRB3^−/−^ old group than in the WT old group (*P* = 0.0005 and 0.0141) (*Figure* 9A and 9B). Moreover, co‐immunoprecipitation experiments demonstrated the binding of TRB3 to p62 in skeletal muscles of young and old mice (*Figure* 9E).

#### TRB3 knockout reduced skeletal muscle fibrosis probably by affecting MAPK signalling pathway

The MAPK signalling pathway was examined by means of western blotting. Relative to the levels in the WT young group, TRB3 expression and JNK and ERK phosphorylation were significantly elevated in the WT old group (*P* = 0.0015, 0.0113, and 0.0007, respectively), whereas p38 phosphorylation exhibited a decreasing tendency (*P* > 0.05). Notably, as compared with the levels in the WT old group, TRB3 expression and JNK phosphorylation were decreased significantly (*P* = 0.0132 and 0.0015), ERK phosphorylation was decreased non‐significantly (*P* > 0.05), and p38 phosphorylation was increased significantly (*P =* 0.0021) in the TRB3^−/−^ old group (*Figure* 9C and 9D). Moreover, co‐immunoprecipitation experiments demonstrated that TRB3 bound to MAPKKs, including MEK1/MEK2, MEK3/MEK6, and MEK4/MKK4 in both young and aged skeletal muscles. However, we detected no direct association between TRB3 and MKK7 (*Figure* 9E).

## Discussion

This study showed that skeletal muscle fibre atrophy, interstitial fibrosis, and decreased motor ability were present in old mice. One of the reasons for these changes could be the high expression of TRB3 in skeletal muscles, which resulted in the inhibition of autophagy and alterations in the MAPK signalling pathway. TRB3 knockout restored the autophagy function, decreased the activation of JNK and ERK, and increased the activation of p38 and thus alleviated the skeletal muscle fibre atrophy, reduced the skeletal muscle fibrosis, and enhanced the exercise capacity of aged mice.

According to the EWGSOP2 diagnostic criteria for sarcopenia, diminished muscle strength is the primary parameter for diagnosing sarcopenia.^19^, ^20^ Here, we recorded a clear decline in old mice of grip strength, inverted hanging time, and exhaustive running time, as well as a reduction in the twitch force and tetanic force of aged EDL muscles. These results indicated decreased muscle strength in the old mice. Furthermore, both the percentage of gastrocnemius weight in body weight and the ratio of gastrocnemius weight to tibia length decreased significantly in old mice, demonstrating obvious sarcopenia. Notably, our study showed that TRB3 was increased in the skeletal muscles of aged mice, which indicated potential TRB3 involvement in the development and progression of sarcopenia, and that TRB3 knockout clearly increased the exercise capacity of old mice, as well as the twitch force and tetanic force of aged muscles. These findings indicate that TRB3 knockout could alleviate sarcopenia in old mice, although the underlying mechanisms remain unclear.

Skeletal muscle fibre atrophy and interstitial fibrosis are the main pathological characteristics during sarcopenia progression. In this study, we found that the CSA of skeletal muscle fibres was decreased in old mice. Intriguingly, the expression of the atrophy‐related E3 ubiquitin ligases atrogin 1 and MuRF1 was markedly elevated in aged skeletal muscles, which suggested increased protein degradation and muscle atrophy. Conversely, the size and the relative content of fast muscle fibres were found to be substantially decreased, which agreed with previous studies showing that fast muscle fibres atrophied and decreased in number earlier than slow muscle fibres.^4^, ^5^, ^6^, ^7^ Our study further showed that TRB3 knockout alleviated skeletal muscle fibre atrophy, which was consistent with the results of previous studies.^13^, ^14^ TRB3 knockout decreased the expression of atrogin 1 and MuRF1 in old mice, which also confirmed that knocking out TRB3 can reduce muscle atrophy. Moreover, our results demonstrated that TRB3 knockout notably alleviated the atrophy of fast muscle fibres and increased the relative content of slow muscle fibres. Slow muscle fibres feature a lower excitation threshold than fast muscle fibres and thus are excited first and generate force during exercise, and when the strength provided by slow muscle fibres cannot meet the demand, excitation of fast muscle fibres begins.^21^ However, fast muscle fibres contain less myoglobin and fewer mitochondria than slow muscle fibres, and fast muscle fibres produce ATP mainly through anaerobic glycolysis and are prone to fatigue during continuous exercise, whereas the opposite is observed with slow muscle fibres.^22^, ^23^ Moreover, relative to fast muscle fibres, slow muscle fibres exhibit stronger ability to contend with and tolerate stimuli such as oxidative stress.^24^ Thus, slow muscle fibres provide support for long‐term exercise endurance. TRB3 knockout might enhance the exercise capacity of aged mice by alleviating atrophy of fast muscle fibres and increasing the relative content of slow muscle fibres.

Various collagens, but mainly collagen I and collagen III, are typically present in skeletal muscles and maintain muscle morphology and function.^25^, ^26^ However, during aging, the content of collagen I and collagen III, particularly that of collagen I, is reported to increase substantially; this then leads to markedly increased tissue stiffness and limited skeletal muscle extension and contraction, which are critical underlying reasons for the decline of skeletal muscle function in aged mice.^9^, ^26^, ^27^ We obtained similar results in our study, and we further found that TRB3 knockout reduced the accumulation of both collagen I and collagen III, with the decrease in collagen I content being greater; these results indicate that TRB3 knockout might exert an anti‐aging effect on skeletal muscles. However, the mechanisms involved here remain to be further elucidated.

Abnormal protein metabolism is a crucial cause of muscle fibre atrophy in aged skeletal muscles,^28^, ^29^ and a key mechanism involved in regulating the homeostasis of protein metabolism is autophagy.^30^ We found here that the autophagy receptor p62 and the autophagosome marker LC3 were increased markedly in aged skeletal muscles, which suggested that autophagy function had clearly declined. Inhibition of autophagy has been reported to potentially aggravate muscle fibre atrophy,^31^ which agrees with our findings. TRB3 could interact with p62 and prevent the binding of p62 to LC3 and ubiquitination substrates, which would lead to the accumulation of p62 and LC3 and inhibition of autophagy/lysosomal degradation.^32^ Accordingly, our study showed a similar result in skeletal muscles, which might account for the accumulation of p62 and the inhibition of autophagy. TRB3 knockout substantially reduced p62 and LC3 levels and increased autophagy function, which probably accounted for the alleviation of skeletal muscle fibre atrophy in aged knockout mice. Therefore, TRB3 knockout might reduce skeletal muscle fibre atrophy by enhancing autophagy.

MAPK signalling could potentially function in fibrous tissue accumulation in skeletal muscles of aged mice. We found that the phosphorylation of ERK and JNK was increased markedly in the muscles of old mice, whereas that of p38 was decreased slightly, and this was accompanied with an increase in interstitial fibrous tissue. Previous clinical studies and animal experiments have yielded consistent results.^33^, ^34^ TRB3 is an upstream regulatory molecule in the MAPK signalling pathway, and a small amount of TRB3 can potently enhance the activation of ERK and JNK and slightly inhibit the activation of p38.^35^, ^36^ Our study revealed that TRB3 interacted with MEK1/MEK2, MEK3/MEK6, and MEK4/MKK4, which can phosphorylate ERK, p38, and JNK and which might account for the activation of MAPK signalling pathway. Notably, TRB3 knockout considerably reduced the phosphorylation of JNK, slightly reduced the phosphorylation of ERK, markedly increased the phosphorylation of p38, and, concurrently, reduced the fibrous tissue content in gastrocnemius muscles of aged mice. These results demonstrated that TRB3 knockout could alleviate skeletal muscle fibrosis by regulating the MAPK signalling pathway.

**Figure 10:**
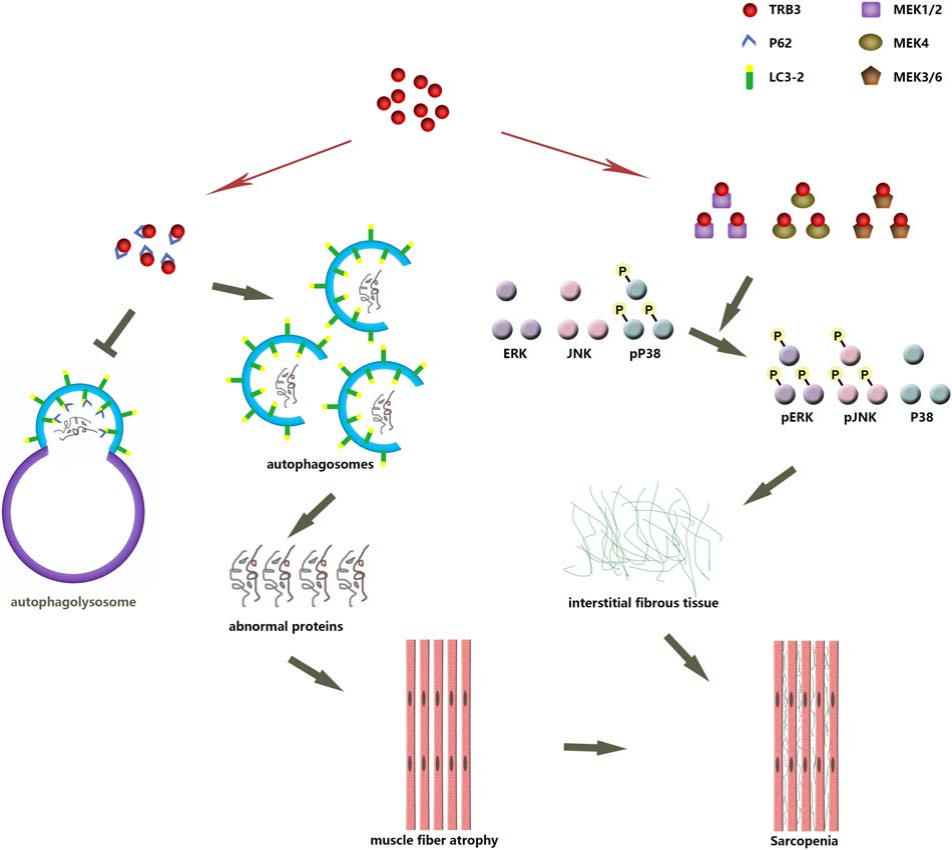
Role of TRB3 in age‐related sarcopenia. In aged skeletal muscles, TRB3 is overexpressed and binds to autophagy receptor p62 to prevent the interaction of p62 with LC3 and ubiquitination substrates and thus inhibits autophagy/lysosomal degradation of abnormal proteins. Accumulation of these harmful proteins triggers muscle fibre atrophy. TRB3 also binds to MAPKKs, including MEK1/MEK2, MEK3/MEK6, and MEK4/MKK4, and leads to increased phosphorylation of ERK and JNK and decreased phosphorylation of p38 and thus causes interstitial fibrosis. Finally, atrophy of muscle fibres and increase of interstitial fibrous tissue lead to sarcopenia.

In conclusion, our results suggest that TRB3 can aggravate age‐related sarcopenia through inhibiting autophagy and activating MAPK signalling pathway, as illustrated in *Figure* 10. TRB3 knockout can alleviate muscle fibre atrophy by enhancing autophagy and reduce skeletal muscle fibrosis by decreasing JNK and ERK phosphorylation and increasing p38 phosphorylation and thereby increase exercise capacity in aged mice and play a protective role in sarcopenia. Our findings thus identify potential intervention targets for the prevention and treatment of age‐related sarcopenia.

## Conflict of interest

None declared.

## Funding

This work was supported by research grants from the National Natural Science Foundation of China (81702194, 81801953, 81873534, 81600633, 81670411, 81570400, 81470560, and 81471036), Key Research and Development Program of Shandong Province (2019GSF108041, 2018GSF118002, 2018GSF118017, and 2017GSF18156), and the Natural Science Foundation of Shandong Province (ZR2019QH010, ZR2014HQ037, and ZR2017BH023).
